# Role of Organochlorine Pesticides in Children with Idiopathic Seizures

**DOI:** 10.1155/2013/849709

**Published:** 2013-12-03

**Authors:** Shilpa Khanna Arora, Prerna Batra, Tusha Sharma, Basu Dev Banerjee, Sushan Gupta

**Affiliations:** ^1^Department of Pediatrics, University College of Medical Sciences (University of Delhi) and GTB Hospital, Delhi 110095, India; ^2^Department of Pediatrics, University College of Medical Sciences and Guru Tegh Bahadur Hospital, Dilshad Garden, Delhi-95, India; ^3^Department of Biochemistry, University College of Medical Sciences (University of Delhi) and GTB Hospital, Delhi 110095, India

## Abstract

*Background*. Organochlorine pesticides (OCP) are persistent organic pollutants that have been implicated in causing several deleterious effects in humans. These are known neurotoxins in high doses, but the role of environmentally acquired OCPs in the body to induce seizures in children has not been investigated yet. *Objectives*. To assess the serum levels of OCPs in children aged 2–12 with idiopathic seizure and to find out any association between the two are our objectives. *Methods*. It was a cross-sectional pilot study. Twenty developmentally normal children aged 2–12, presenting with idiopathic generalized seizures, were recruited. Twenty age-matched controls without any history of seizures were also taken. Their serum levels of *α*, *β*, and *γ* hexachlorocyclohexane (HCH); and aldrin; dieldrin; p,p-dichlorodiphenyltrichloroethane (DDT), o,p-DDT, and p,p dichlorodiphenyldichloroethylene (DDE); and *α* and *β* endosulfan were analysed using gas chromatography (GC). Mann-Whitney *U* test was used to compare OCP levels between the groups. Spearman correlation was used to find the correlation between individual pesticide levels with age and seizure duration. *Results*. Levels of *β*, *γ*, and total HCH were significantly higher among cases as compared to the control group (*P* ≤ 0.05). *Conclusion*. There exists a possible association between idiopathic seizures and high serum levels of OCPs, especially HCH.

## 1. Introduction

Organochlorine pesticides (OCPs) are amongst the most commonly used pesticides in developing countries because of their low cost and broad spectrum of activity against various pests. India hosts one of the largest pesticide manufacturing industries in the world, producing around 90,000 metric tons of pesticides every year [1].

OCPs are persistent organic pollutants (POPs) that are toxic and bioaccumulative in nature [2, 3]. These pesticides not only tend to accumulate in adipose tissue but also biomagnify through food chain due to their lipophilic nature and long half-lives [4]. They have a wide range of acute and chronic health effects like cancer, reproductive disorders, immune suppression, congenital defects, and endocrine dysfunction [5–7]. Hexachlorocyclohexane (HCH) isomers including lindane (*γ*-HCH) are anticipated to be human carcinogens based on animal experiments that have generated sufficient evidence of carcinogenicity [5]. Many cases of human aplastic anemia, leukemia, and lung cancer have also been reported in association with HCH exposure. Prenatal exposure to HCH has been implicated in causing thyroid dysfunction in children [6]. Transplacental transfer of OCPs, especially HCH, has been found to be associated with low birth weight in neonates [7].

Organochlorine pesticides have also been proven to have several deleterious effects on the central nervous system [8]. OCP exposure has been implicated in causing neurobehavioral deterioration, cognitive dysfunction, dementia, and so forth [9–16]. Studies have associated pesticide exposure with Parkinsonism and Alzheimer's disease as well [9–12]. It has also been postulated that exposure of pesticides to the developing human brain prenatally as well as through breast milk can result in subtle negative effects on neurological and cognitive development of the children in later life [13–16]. These chemicals are known to produce their CNS effects by disturbing the process of neurotransmission in the brain.

Several OCPs like lindane, that is, gamma-hexachlorocyclohexane (*γ*-HCH) and endosulfan have been demonstrated to induce seizure in animal models [17, 18]. There are reports of seizures after organochlorine pesticide toxicity in humans, but the role of environmentally acquired OCPs present in the body to induce seizures in children has not been investigated yet. Thus, the present study was carried out to measure the serum levels of OCP in children aged 2–12 with idiopathic seizures, with the hypothesis that raised levels of organochlorine pesticides may have been responsible for inducing seizures in patients with idiopathic seizures and normal neuroimaging.

## 2. Subjects and Methods

The study was a cross-sectional pilot study conducted in the departments of pediatrics and biochemistry of a tertiary care teaching hospital of Delhi, India. Ethical clearance was obtained from the institutional ethical committee prior to the initiation of this study.

### 2.1. Inclusion and Exclusion Criteria

Twenty consecutive developmentally normal children aged 2–12, presenting to the hospital with generalised seizures within the last 24 hours and having a normal neuroimaging (contrast enhanced computed tomography or magnetic resonance imaging), were recruited in the study. Children who were already receiving antiepileptic drugs or having a known exposure to any toxin/chemical or suffering from any endocrine/hepatic/renal or allergic disorders were excluded from the study. A total of 20 age-matched controls without any history of seizures and who had been admitted in ward with an unrelated complaint were recruited as controls. Written informed consent was taken from the parent/guardian of all the cases and controls.

### 2.2. Data Recording

A detailed case record form was filled up for each of the subjects. The details included the demographic profile; socioeconomic status as per modified Kuppuswamy scale [20]; occupation; diet (vegetarian or nonvegetarian or exposure to junk food); drinking water supply (tap water or ground water); any recent or past exposure to pesticides; any exposure to passive smoking; detailed history of seizure type and duration and number of episodes; and detailed general physical as well as systemic examination.

### 2.3. Sample Collection, Storage, and Processing

Venous blood samples were collected within 24 hours of seizure from the cases. Three to five millilitres of venous blood was withdrawn from each of the subjects and stored. The process of extraction and quantification of pesticides was carried out in the department of biochemistry in a blinded manner. Extraction of *α*, *β*, and *γ* HCH; aldrin; dieldrin; p,p-DDT; o,p-DDT, and p,p-DDE; *α* and *β* endosulfan was done using gas chromatography (GC), grade hexane and acetone (2 : 1) according to method of Bush et al. [19]. All the solvents used for OCP extraction were of HPLC grade and free from any contamination. OCPs extraction was done using hexane and acetone (2 : 1) according to the previously described method [19] with minor modifications. Hexane and acetone (in ratio of 2 : 1) were added to one mL of blood and the whole mixture was shaken at room temperature for 30 min in a mechanical shaker. The extract was centrifuged at 2000 rpm for 10 min and clear top layer of hexane was collected. The remaining portion was again extracted twice using the same process and the newly extracted hexane layer was added to the previous solvent fractions. Cleanup of the samples was done by column chromatography following USEPA method 3620B. Elute was collected, concentrated, and redissolved in hexane for further analysis. Quantification of OCP levels was done by Perkin Elmer gas chromatograph (GC) equipped with ^63^Ni selective electron capture detector. The column used was Elite-GC DB-5, 60 meter, and 0.25 mm ID. The carrier and makeup gas was nitrogen with a flow rate of 2 mL min^−1^ and 35 mL min^−1^, respectively, employing the splitless mode. Final extract (1 *μ*L) was injected at a temperature of 170°C with a hold time of 1 min. The temperature was raised from 170°C to 225°C at a rate of 5°C min^−1^ with a hold time of 5 min and finally from 225°C to 275°C at a rate of 6°C min^−1^ with a hold time of 15 min. The total run time was 40 min per sample.

Quantitative analysis of OCP residues of each sample was done by comparing the peak area with those obtained from a chromatogram of a mixed OCPs standard (Supelco, Sigma—Aldrich) of known concentration. Analytes were confirmed by spiking with known standards of pesticides (Supelco, Sigma—Aldrich). The detection limit of the detector was 0.05 pg perchloroethylene with nitrogen as a carrier gas. The detection limit of the method was 4 pg mL^−1^ for each OCP. For quality control, five blood samples in triplicate were spiked with a mixed standard of OCPs at 5 and 25 ng mL^−1^. The average recoveries of fortified samples exceeded 95%. The case and control samples were run in the same analytical batches and for quality check, and a sample was always run with each set of samples for pesticide analysis to maintain accuracy. For the internal control of our measurements, pesticide identification was confirmed by GC-MS at Central Pollution Control Board, New Delhi, Ministry of Environment and Forests, Government of India, under our collaborative research programme as per MOU guidelines between two institutes. The levels of pesticides were expressed as parts per billion (ppb).

### 2.4. Statistical Analysis

Statistical analysis was done using SPSS Version 20.0, SPSS Inc., Chicago. The levels of individual pesticides were expressed as mean and median. Inter- and intragroup Comparisons of median pesticide levels were done using Mann-Whitney *U* test. Spearman correlation was used to find the correlation between individual pesticide levels with age and seizure duration.

## 3. Results

A total of 20 cases and 20 controls were studied whose baseline data has been summarised in Table 1. The baseline characteristics of cases and controls were comparable. Our hospital caters mostly to population residing in urban slum and there are no pesticide plants in the area. None of the patients had history of exposure to pesticides and none were into farming. The majority of subjects belonged to middle or lower socioeconomic strata as per modified Kuppuswamy scale. Out of twenty, eight patients had a single episode of seizure at enrolment and another eight had seizures lasting for >15 minutes.

The mean ± SD and median (interquartile range) levels of the individual pesticides amongst cases and controls have been given in Table 2. Levels of *β*, *γ*, and total HCH were significantly higher among cases as compared to control group (*P* values <0.000, =0.014, and <0.000). The level of *α* endosulphan was also high in cases as compared to that in controls, though it did not reach statistical significance.

The serum levels of pesticides in either of the groups did not demonstrate any correlation with age or seizure duration. There was no significant difference in the serum level of any of the pesticides with the type of diet, consumption of junk food, and passive smoking.

## 4. Discussion

We observed significantly higher levels of *β*, *γ*, and total HCH in the seizure patients in comparison to controls. Seizures in children occur due to reduced convulsive threshold, which may be due to an organic cause or may be idiopathic. EEG recorded seizures have been observed in OCP intoxication which often get masked in the presence of the typical flaccid paralysis due to neuromuscular junction blockage [21]. Higher levels of *β*, *γ*, and total HCH point towards organochlorine pesticides having some association with idiopathic seizures in this age group.

Gamma HCH (active component of lindane) is well known to induce seizure in mammals [17, 22, 23]. Fishman has demonstrated in mice that the actions of the HCH isomers in the CNS appear to be mediated through GABA-A receptor linked chloride channel sites which in turn results in cerebellar cyclic GMP accumulation [24]. Lindane is used mainly as an insecticide for fruit and vegetable crops; for treatment of wood, seed grains, and livestock; and in baits for rodent control. For humans, the only use is as a second-line pharmaceutical treatment for lice and scabies. The use is approved by the U.S. Food and Drug Administration in some scabicidal and antilice products [25]. WHO classifies lindane as “moderately hazardous,” and its international trade is restricted and regulated under the Rotterdam Convention on prior informed consent [26]. Internationally, the production and agricultural use of lindane was banned in 2009 under the Stockholm Convention on persistent organic pollutants. India is one of the few countries in the world where restricted usage of lindane was allowed until recently.

Our study also observed higher blood levels of *α* and *β* endosulfan in the patients of idiopathic seizures as compared to controls, though the levels were not statistically significant, possibly due to a small sample size. Endosulfan has been used extensively in agriculture as an insecticide for managing resistance due to its unique mode of action. It is a potentially neurotoxic pesticide. Gilbert has demonstrated that repeated administration of subconvulsive doses of lindane as well as endosulfan tends to induce seizure by lowering seizure threshold by binding to GABA receptor ionophore complex in amygdale kindled animals [18]. There are reports of acute seizures in humans, induced by endosulfan, in absence of other features of OCP intoxication. Kutluhan has reported acute repetitive seizures of uncertain etiology in three young subjects, without any other sign of intoxication, which were later attributed to suicidal consumption of relatively small amount of endosulfan [27]. World Health Organization classifies it as “moderately hazardous” [26]. Endosulfan usage is being phased out globally following the Stockholm Convention in April 2011, but it is still used extensively in India.

Other OCPs like DDT, aldrin, and dieldrin are also known to induce seizures in mammals by affecting different neuronal pathways [28–30]. DDT has been postulated to produce stimulatory effect on CNS by increasing the concentration of free ammonia which may be involved in reducing the level of GABA [28]. Aldrin tends to reduce CNS excitability threshold for seizure, the effects mediated by noradrenergic pathways [30]. We could not demonstrate significantly higher levels of these pesticides in our study. Dichlorodiphenyltrichloroethane (DDT) is banned in India for use in agriculture since 1998 but is still used for vector control [31]. Also, other OCPs like aldrin, endrin, and dieldrin have been banned for manufacture, import, as well as use in the country.

To the best of our knowledge, this is the only study, where high serum levels of *β*, *γ*, total HCH, and endosulfan have been found to be possibly associated with idiopathic seizures in children. The study raises the concern of an association or a possible causation between environmentally acquired raised serum OCP levels and seizures of unproven etiology in children. Ours is a pilot study with a small sample size. We suggest the need to carry out large-scale, multicentric, and longitudinal studies to further establish an association or a possible causation between serum OCP levels and seizures of unproven etiology in children. Also the public health authorities in India need to make the laws relating to manufacture, usage, and disposal of OCPs much more stringent.

## Figures and Tables

**Table 1:** Baseline characteristics of subjects.

	Cases [20]	Controls [20]
Sex distribution		
Male	13	15
Female	7	5
Age distribution (years)		
Mean ± SD	4.8 ± 2.6	4.75 ± 2.69
Diet		
Vegetarians	8	5
Nonvegetarians	12	15
Junk diet		
Yes	17	18
No	3	2
Water supply		
Tap water	19	17
Ground water	1	3
Passive smoke exposure		
Yes	6	10
No	14	10

**Table 2:** Pesticide levels (ppb) in cases and control groups.

Pesticide	Cases			Controls			*P* value
	Mean ± SD	Median	Interquartile range (25th–75th centile)	Mean ± SD	Median	Interquartile range (25th–75th centile)	
*α*-HCH	4.97 ± 2.04	5.06	3.29–6.30	4.19 ± 1.652	4.07	3.41–5.49	0.237
*β*-HCH	8.90 ± 4.69	8.18	5.24–12.36	2.70 ± 1.71	2.71	1.24–3.75	0.000*
*γ*-HCH	5.05 ± 3.09	4.79	2.40–6.18	2.89 ± 1.71	3.35	1.79–4.17	0.014*
T-HCH	18.92 ± 8.04	17.42	14.61–22.89	9.77 ± 3.40	9.27	7.57–12.81	0.000*
Aldrin	1.49 ± 1.75	0.60	0.00–3.12	1.97 ± 1.352	1.84	1.20–2.72	0.362
Dieldrin	1.81 ± 2.36	1.14	0.00–3.69	1.75 ± 1.42	2.33	0.00–3.00	0.661
P,P DDT	1.09 ± 1.25	0.80	0.00–2.12	1.55 ± 0.88	1.39	1.16–2.38	0.077
P,P DDE	3.13 ± 2.53	3.24	1.13–4.62	3.79 ± 1.66	3.65	2.53–4.90	0.273
*α* Endosulfan	1.42 ± 1.21	1.69	0.00–2.61	0.89 ± 0.76	0.91	0.00–1.30	0.124
*β* Endosulfan	1.04 ± 1.07	1.02	0.00–2.04	0.80 ± 0.69	0.92	0.00–1.31	0.474

*P value < 0.05.

## References

[1] Abhilash PC, Singh N (2009). Pesticide use and application: an Indian scenario. *Journal of Hazardous Materials*.

[2] Kannan K, Tanabe S, Tatsukawa R (1995). Geographical distribution and accumulation features of organochlorine residues in fish in tropical Asia and Oceania. *Environmental Science and Technology*.

[3] Shegunova P, Klánová J, Holoubek I (2007). Residues of organochlorinated pesticides in soils from the Czech Republic. *Environmental Pollution*.

[4] Siddiqui MKJ, Srivastava S, Srivastava SP, Mehrotra PK, Mathur N, Tandon I (2003). Persistent chlorinated pesticides and intra-uterine foetal growth retardation: a possible association. *International Archives of Occupational and Environmental Health*.

[5] NTP (2011). *Report on Carcinogens*.

[6] Álvarez-Pedrerol M, Ribas-Fitó N, Torrent M (2008). Thyroid disruption at birth due to prenatal exposure to *β*-hexachlorocyclohexane. *Environment International*.

[7] Dewan P, Jain V, Gupta P, Banerjee BD (2013). Organochlorine pesticide residues in maternal blood, cord blood, placenta, and breast milk and their relation to birth size. *Chemosphere*.

[8] Wang X, Wang D, Qin X, Xu X (2008). Residues of organochlorine pesticides in surface soils from college school yards in Beijing, China. *Journal of Environmental Sciences*.

[9] Abhilash PC, Singh N (2009). Pesticide use and application: an Indian scenario. *Journal of Hazardous Materials*.

[10] Fleming L, Mann JB, Bean J, Briggle T, Sanchez-Ramos JR (1994). Parkinson’s disease and brain levels of organochlorine pesticides. *Annals of Neurology*.

[11] Corrigan FM, Murray L, Wyatt CL, Shore RF (1998). Diorthosubstituted polychlorinated biphenyls in caudate nucleus in Parkinson’s disease. *Experimental Neurology*.

[12] Richardson JR, Shalat SL, Buckley B (2009). Elevated serum pesticide levels and risk of Parkinson disease. *Archives of Neurology*.

[13] Boersma ER, Lanting CI (2000). Environmental exposure to polychlorinated biphenyls (PCBs) and dioxins: consequences for longterm neurological and cognitive development of the child lactation. *Advances in Experimental Medicine and Biology*.

[14] Gladen BC, Rogan WJ, Hardy P, Thullen J, Tingelstad J, Tully M (1988). Development after exposure to polychlorinated biphenyls and dichlorodiphenyl dichloroethene transplacentally and through human milk. *Journal of Pediatrics*.

[15] Rogan WJ, Gladen BC (1991). PCBs, DDE, and child development at 18 and 24 months. *Annals of Epidemiology*.

[16] Koopman-Esseboom C, Weisglas-Kuperus N, De Ridder MAJ, Van Der Paauw CG, Tuinstra LGMT, Sauer PJJ (1996). Effects of polychlorinated biphenyl/dioxin exposure and feeding type on infantś mental and psychomotor development. *Pediatrics*.

[17] Fishman BE, Gianutsos G (1988). CNS biochemical and pharmacological effects of the isomers of hexachlorocyclohexane (lindane) in the mouse. *Toxicology and Applied Pharmacology*.

[18] Gilbert ME, Mack CM (1995). Seizure thresholds in kindled animals are reduced by the pesticides lindane and endosulfan. *Neurotoxicology and Teratology*.

[19] Bush B, Snow J, Koblintz R (1984). Polychlorobiphenyl (PCB) congeners, p,p’-DDE, and hexachlorobenzene in maternal and fetal cord blood from mothers in upstate New York. *Archives of Environmental Contamination and Toxicology*.

[20] Mishra D, Singh HP (2003). Kuppuswamy’s socioeconomic status scale—a revision. *Indian Journal of Pediatrics*.

[21] Tattersall J (2009). Seizure activity post organophosphate exposure. *Frontiers in Bioscience*.

[22] Vucević D, Hrncić D, Radosavljević T (2008). Correlation between electrocorticographic and motor phenomena in lindane-induced experimental epilepsy in rats. *Canadian Journal of Physiology and Pharmacology*.

[23] Stark LG, Joy RM, Hollinger MA (1987). Effects of two isomers of hexachlorocyclohexane (HCH) on cortical *β*-adrenoceptors in rat brain. *Experimental Neurology*.

[24] Fishman BE, Gianutsos G (1987). Opposite effects of different hexachlorocyclohexane (lindane) isomers on cerebellar cyclic GMP: relation of cyclic GMP accumulation to seizure activity. *Life Sciences*.

[25] Lindane http://www.fda.gov/downloads/Drugs/DrugSafety/UCM133687.

[26] World Health Organization (2009). *The WHO Recommended Classification of Pesticides by Hazard and Guidelines to Classification*.

[27] Kutluhan S, Akhan G, Gultekin F, Kurdoglu E (2003). Three cases of recurrent epileptic seizures caused by Endosulfan. *Neurology India*.

[28] Matin MA, Jaffery FN, Siddiqui RA (1981). A possible neurochemical basis of the central stimulatory effects of pp’DDT. *Journal of Neurochemistry*.

[29] Albertson TE, Joy RM, Stark LG (1985). Chlorinated hydrocarbon pesticides and amygdaloid kindling. *Neurobehavioral Toxicology and Teratology*.

[30] Castro VL, Palermo-Neto J (1989). Effects of long-term aldrin administration on seizure susceptibility of rats. *Pharmacology & Toxicology*.

[31] Kumar A, Dayal P, Shukla G, Singh G, Joseph PE (2006). DDT and HCH residue load in mother's breast milk: a survey of lactating mother's from remote villages in Agra region. *Environment International*.

